# Nanotribological Properties of Oxidized Diamond/Silica Interfaces: Insights into the Atomistic Mechanisms of Wear and Friction by Ab Initio Molecular Dynamics Simulations

**DOI:** 10.1021/acsanm.3c02881

**Published:** 2023-09-04

**Authors:** Huong Thi Thuy Ta, Nam Van Tran, Maria Clelia Righi

**Affiliations:** Department of Physics and Astronomy, University of Bologna, 40127 Bologna, Italy

**Keywords:** friction, diamond wear, atomistic mechanisms, ab initio molecular dynamics, tribochemical reactions

## Abstract

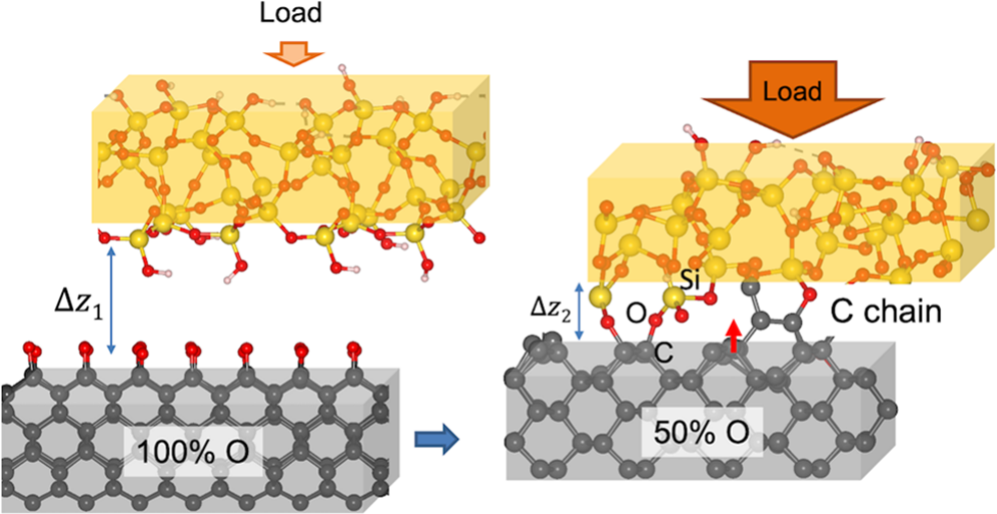

Controlling friction and wear at silica–diamond interfaces is crucial for their relevant applications in tribology such as micro-electromechanical systems and atomic force microscopes. However, the tribological performance on diamond surfaces is highly affected by the working environment where atmospheric gases are present. In this work, we investigate the effects of adsorbed oxygen on the friction and wear of diamond surfaces sliding against silica by massive ab initio molecular dynamics simulations. Different surface orientations, O-coverages, and tribological conditions are considered. The results suggest that diamond surfaces with full oxygen passivation are very effective in preventing surface adhesion, and as a result present extremely low friction and wear. At low oxygen coverage, Si–O–C bond formation was observed as well as atomistic wear initiated from C–C bond breaking at extreme pressure. The analysis of electronic structures of the configurations resulting from key tribochemical reactions clarifies the mechanisms of friction reduction and atomistic wear. Overall, our accurate in silico experiments shed light on the influence of adsorbed oxygen on the tribological properties and wear mechanisms of diamond against silica.

## 1 Introduction

Due to its outstanding stability and hardness, diamond has been renowned as an ideal coating material for ultra-precision manufacturing, cutting tools, micro-electromechanical systems (MEMS), and atomic force microscopes (AFM).^1−3^ In these applications and devices, silica–diamond interfaces can be present and play a key role responsible for load bearing and wear resistance at nanoscale contacts. However, the tribological properties of diamond are deeply affected by extrinsic factors, including surface termination upon interactions with adsorbed species during fabrication,^4^ etching,^5^ and working environment.^6−8^ Thus, studies of friction and wear of passivated diamond are intensively carried out for their practical applications. Among them, oxygenation is one of the most common superficial reactions of diamond due to the dissociation of molecular oxygen^7,9,10^ and OH dehydrogenation in tribological conditions or at elevated temperatures,^11^ making oxygen termination an essential feature of diamond surfaces.^10^ The presence of oxygen can, in fact, significantly modify the diamond surface chemistry and its electronic structure,^10^ altering its reactivity,^9^ electron affinity,^12^ and key electrical/photonic properties.^13,14^ Understanding the nanoscale friction and wear mechanisms of the oxidized diamond is, thus, highly relevant to optimize its full potential in a wide range of applications and technologies.^15−18^

Various mechanisms have been reported to explain the ultralow friction and wear at diamond–diamond interfaces.^16,19,20^ One key mechanism is the passivation of reactive dangling bonds, which effectively reduces adhesive friction at diamond sliding contacts.^15,19,21,22^ So far, hydrogen and oxygen have been recognized as effective passivating species for friction reduction at diamond–diamond contacts.^18^ Apart from surface adsorbed species, the concentration of passivating agents^17^ and the nature of counter surfaces^18,23^ can profoundly influence the friction and wear performance of diamond. Wang et al. have proposed that oxygen coverage greater than 0.5 is necessary to effectively prevent chemical bonding at diamond–diamond interfaces.^17^ When it comes to silica–diamond contacts, O-terminated diamond surfaces exhibit lower adhesion and larger separation compared to H-passivated surfaces, mainly due to the large atomic size of oxygen, which induces higher steric hindrance compared to hydrogen.^23^ The presence of C–O and C=O functional groups can alter the diamond surface chemistry and its reactivity.^9,10^ As oxygen is dissociated on diamond at a lower energy barrier than those of H_2_ and H_2_O,^24^ the oxygenation of diamond surfaces tends to be more prevalent than hydrogenation and hydroxylation. However, the atomistic understanding of the effects of oxygenation on the trbilogical properties of silica–diamond interfaces remains limited.

In addition to friction, the surface rubbing at high applied loads can initiate wear. Studies on diamond-like carbon films indicated that wear can be formed through the transformation from diamond-like to graphite-like carbon,^25^ which later was verified by molecular dynamics (MD) simulations by a pilot atom concept.^26,27^ Furthermore, wear can be initiated under different forms of carbon including carbon chains,^28^ atom-by-atom, and carbon sheets.^29^ Under oxygenation, carbon removal can proceed through the desorption of CO or CO_2_,^8,10,30^ suggesting the essential role of oxygen in the formation of wear. Interestingly, the wear of diamond surfaces was observed when sliding against softer materials such as silica or silicon.^26,31−35^ In these systems, the chemical adhesion and wear are initiated by the Si–C/C–O–Si chemical bonds between two mating surfaces.^26^ Thus, a systematic study considering different coverage concentrations, surface orientations, and tribological conditions is of significance to shed light on the wear of oxygenated diamond sliding against silica.

In this work, we performed ab initio molecular dynamics (AIMD) simulations to mimic the tribological conditions of silica sliding against diamond surfaces. The full quantum mechanical description allows an accurate description of the chemical reactions occurring in conditions of enhanced reactivity as those present in the tribological contacts. We consider the three most common low-index diamond surfaces, i.e., the C(001), C(110), and the Pandey reconstructed C(111) (*R*-C(111)) surfaces. Two different oxygen coverages of 50 and 100% were modeled to account for the partial or total passivation of the surface. The counter surface of silica with an amorphous structure was used to model silicon oxide that is commonly formed when silicon is exposed to air in many technical applications such as micro-electromechanical systems. The simulations were performed at 1 GPa to investigate the frictional performance of oxygen-passivated diamond surfaces and compare it with hydrogen-passivated ones under the same tribological conditions.^23^ AIMD simulations at a hasher condition were also performed to activate wear, the atomistic mechanisms of which have been explained on the basis of the electronic structure analysis.

The presented study relies on fully ab initio simulations, which ensure an accurate description of the bond breaking and forming events activated under tribological conditions. Large-size models (up to ∼400 atoms) are adopted for a realistic description of the amorphous silica surface, and long simulation runs (15 ps for each run) are performed for several systems in parallel. The computational effort spent for the production of the presented results puts our work at the frontier of what can be currently done with massive fully ab initio calculations. We expect that the outcomes of our simulations will be relevant for enhancing the understanding of the processes occurring during the silica-driven polishing of diamond surfaces.

## 2 Simulation Method and Models

AIMD simulations were performed using a modified version of the Quantum Espresso package,^36^ which allows simulating the tribological conditions. The code has been successfully used for studying the tribochemistry of different systems, including diamond–silica interfaces.^21,23^ The generalized gradient approximation (GGA) with the Perdew–Burke–Ernzerhof (PBE)^37^ method was used as the exchange–correlation functional. The plane-wave basis set was used to expand the electronic wave function, and the core electrons were represented by ultrasoft pseudopotentials with the cutoff energies of 30 Ry for the wave function and 240 Ry for the charge density, respectively. The Γ-point was used for the Brillouin zone sampling to compromise the computational cost considering the large models and a long simulation time of 15 ps. A semiempirical correction by Grimme (D2)^38,39^ was adopted to account for the long-range van der Waals interactions. The Verlet algorithm with a timestep of 20 atomic units (au) of time, corresponding to ∼1 fs was used.

The amorphous silica was built as technically described in our previous work.^23^ The surface termination contains sixloxane (Si–O–Si) and silanol (Si–OH) groups as a result of silicon oxide in contact with the environment. In practice, silica surface can be terminated by several types of silanols such as isolated, vincinal, and geminal silanols which present different chemistry and affect the tribological properties of silica. The geminal silanols are rare and thus their contribution is relatively small compared to isolated and vicinal silanols.^40^ Within the scale of quantum simulations, it is challenging to reproduce the exact chemistry of the amorphous silica surface. Nevertheless, the silica model used in this work has been carefully constructed and its termination was chosen in order to reproduce the silanol density observed in experiments; see details in a previous work of our group.^23^ Three diamond surfaces with two different oxygen concentrations and the same sizes as silica surface are mated against silica, for a total of six silica–diamond interfaces. Details about the sizes of the simulation systems are reported in Table 1. The initial structures of these silica–diamond systems are reported in Figure S2 in the Supporting Information (SI).

**Table 1:** Number of Atoms, Thickness, and Dimensions of Silica–Diamond Systems Simulated in This Work

system	dimension (Å^2^)	no. atom (silica)	no. atom (diamond)^a^	no. C layer
silica–C(110)	10.12 × 17.88	120	210/220	4
silica–C(001)	10.12 × 17.88	120	238/252	5
silica–*R*-C(111)	10.12 × 8.94	60	152/160	5

a The first and second numbers are reported for 50 and 100% oxygen coverage, respectively.

An external force corresponding to a load of 1 GPa was applied along the *z*-direction on the Si atoms at the topmost layer. The pressure of 1 GPa was applied as a typical load to study tribological properties and to allow for comparison with results obtained in a previous work on (partially) hydrogenated diamond surfaces sliding against silica performed by our group.^23^ The sliding conditions were modeled by using a modified version of the Born Oppenheimer molecular dynamics implemented in the Quantum Espresso package,^21^ which prevents the effect of the thermostat on the sliding motion.^21^ A constant vertical load was imposed by applying vertical forces on the Si atoms of the topmost layer of the silica slab, while the bottommost layer of the diamond slab was kept fixed. The temperature was controlled at 300 K by rescaling the atoms’ velocities. After the relaxation under load and equilibration at a temperature of 300 K, the sliding motion is started by imposing a constant velocity of 200 m/s to the topmost Si atoms of the silica slab along the *x*-direction. Detailed description of the load and velocity control by the modified code can be found in Zilibotti et al.^21^ It is worth mentioning that under the local contact, the contact between asperities can cause flash temperature/pressure leading to extreme condition where temperature and pressure can reach 1000 °C and 10 GPa.^41,42^ Therefore, In order to facilitate the formation of wear, the simulations were also performed at harsh conditions of 10 GPa and 600 K to represent extreme working conditions, where chemical processes such as C–C bond breaking can be activated in the simulated time interval. The frictional properties of the systems were analyzed by calculating the interfacial distance between the silica and diamond surfaces and the resistive forces. The interfacial distance was calculated by the subtraction of the average *z* coordinates of Si and C atoms at the interfacial layers. The resistive forces are the sum of the *x* component of Si atoms to which the external forces and velocity constraints were applied.^21,23^ The average values and errors of the interfacial distance and forces were estimated by using the block average procedure as reported by Templeton et al.^43^ The movies of the AIMD simulations of silica sliding against oxidized diamond surfaces are provided as a part of the Supporting Information.

To understand the nature of the silica–diamond interaction, the perpendicular potential energy surfaces (P-PES) which measure the adhesion between the two surfaces as a function of interfacial distance were calculated. To estimate the errors, the adhesion energies were calculated for four different lateral positions of silica on diamond surfaces following eq 1
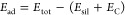
1where *E*_tot_, *E*_sil_, and *E*_C_ are the energies of the silica–diamond system, silica, and diamond surfaces, respectively. The lowest one was utilized to describe the P-PES while the others were used to estimate the associated error bars calculated as the difference between the selected P-PES and the average value of the other three. To make a comparative conclusion about the effect of oxygen and hydrogen passivation on the frictional properties of the silica–diamond systems, the P-PESs were calculated for both O- and H-passivated diamond surfaces.

The electronic structure of selected configurations related to interfacial reactions was explored in terms of Bader charges and bond overlap population (BOP) analyses. The Bader charges are calculated by partitioning the charge density into Bader volumes and separating atoms based on a zero flux surface.^44^ The BOP which measures the overlap population between two selected atoms was calculated by the Lobster code.^45^

## 3 Results and Discussion

### 3.1 Diamond Surfaces

**Figure 1:**
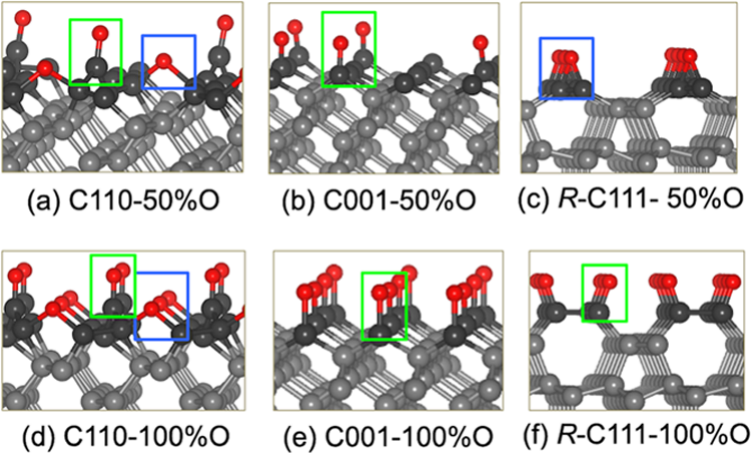
Optimized structures of the 50 and 100% oxygen terminated C(110) (a, d), C(001) (b, e), and *R*-C(111) (c, f) surfaces. Color assignment: O (red), C (gray). The darker balls show the carbon atoms at the top layer. The green and blue boxes mark the carbonyl and epoxy configurations of the diamond surfaces. Top and side views of the corresponding diamond surfaces are shown in Figure S1.

The optimized structures of the six diamond surfaces studied in this study are presented in Figure 1. These surfaces include the C(001), C(110), and *R*-C(111) surfaces covered with oxygen in two different concentrations of 50 and 100%. The oxygenation of the diamond surfaces is attributed to dissociative adsorption of oxygen, forming ether or ketone configurations depending on the surface orientation and the oxygen coverage.^8−10,46^ In particular, the ketone configuration dominates on the C(001) surface at both 50 and 100% coverages.^9,10^ For the *R*-C(111) surface, the ether configuration is more favorable at low coverage, while the ketone group prevails at higher coverage.^8^ On the C(110) surface, there is a coexistence of the ether and the ketone groups (Figure 1).^46^ Active carbon sites are present on only C110-50%O and C001-50%. The former contains fewer active carbon sites than the latter due to the combination of the ketone and ether configurations. The surface configurations as well as the oxygen coverage play a crucial role in governing the surface chemistry and the reactivity of diamond in dynamics tribological simulations.

### 3.2 Tribological Properties of Silica-Oxidized Diamond Interfaces

**Figure 2:**
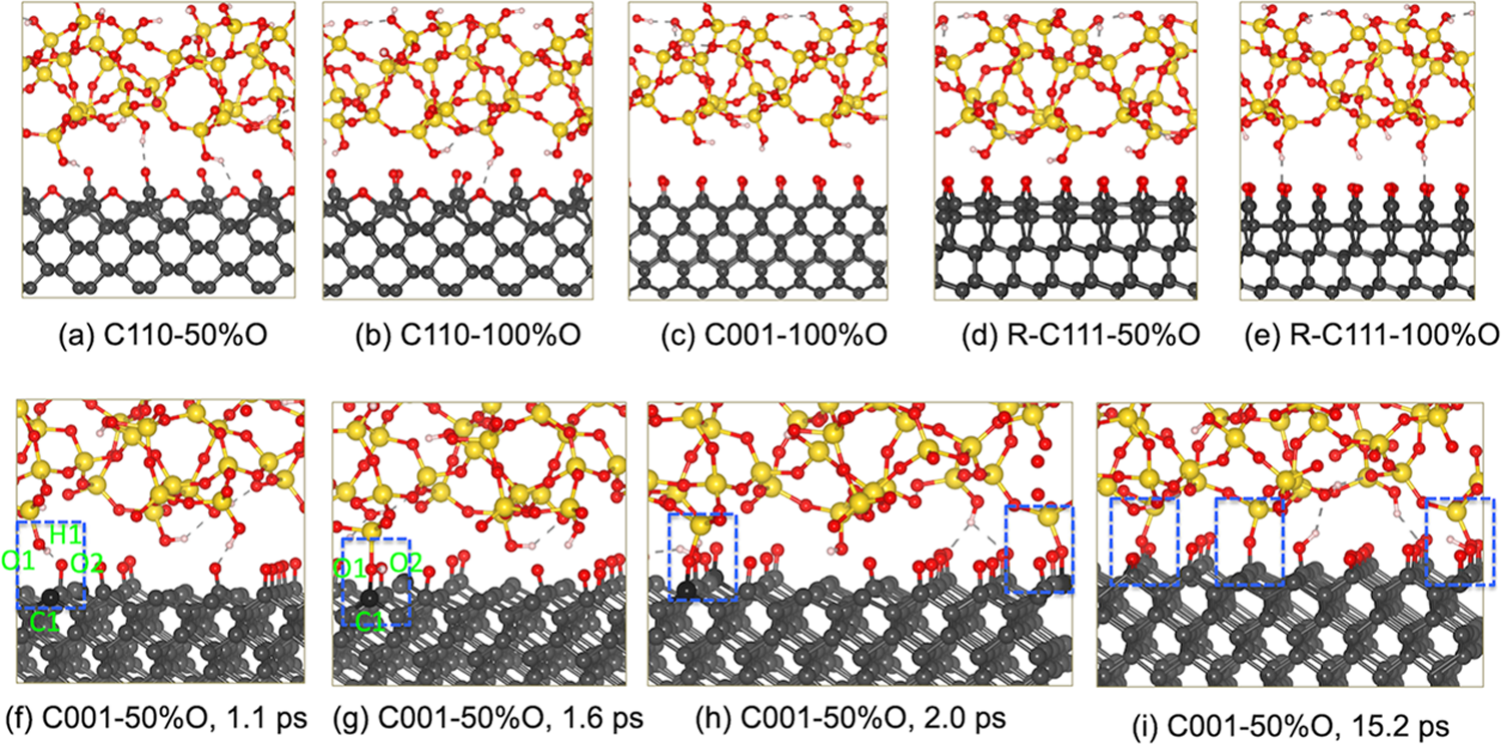
Snapshots of chemical events occurring at the silica–diamond interface during the tribochemical simulation at 1 GPa and 300 K. Color assignment applied throughout this work: O (red), C (gray), Si (yellow), H (white). The darker balls show the carbon atoms evolving in the chemical reactions with silica. From (a) to (e), snapshots at 10 ps, hydrogen bonds forming at the interfaces of silica and C(110), C001-100%O, and *R*-C(111) surfaces. (f) Hydrogen bond between O2 and H1, (g) Si–O–C bond formation, and (h, i) additional Si–O–C bonds formed at the interface.

The relaxation and equilibration of the six silica–diamond systems were performed under a load of 1 GPa and a temperature of 300 K for 1 ps before initiating the sliding simulations for a duration of 15 ps. The structures of the six simulated systems after the relaxation at 1 GPa are depicted in Figure S2, which feature the hydrogen bonds formed between H of the silanol groups and O of the diamond across the interfaces. Snapshots of the atomic structures during the AIMD sliding simulations of the silica–diamond systems are shown in Figure 2. During the sliding at 1 GPa, except for the C001-50% system, no chemical bonds are formed in other systems (Figure 2a–e and Movies S1 and S2). This result clearly demonstrates that 50 and 100% oxygen passivation effectively prevents the chemical bond formation across the silica–diamond interface. The chemical bonds are observed only in the C001-50%O system, where OH bond dissociation followed by Si–O–C bond formation occurs, making this system the most reactive one at 1 GPa. The reactions on C001-50%O system occur in a three-step process (Figure 2f–i and Movie S3): (1) The formation of the hydrogen bond between H1 of the silica and O2 of the carbonyl groups on the C(001) surface; (2) the dissociation of the O1–H1 bond, leading to the formation of the new O2–H1 bond on the C(001) surface and leaving a nonbridging oxygen atom O1; and (3) the newly formed nonbridging oxygen becomes chemically active, facilitating the formation of the C1–O1–Si bridge at the silica–C(001) interface (Figure 2g). During sliding, the relative movement of the silica results in the stretching and dissociation of the Si–O bonds. Despite the chemical interactions observed in this system, the sliding primarily results in the breaking of Si–O bonds rather than C–O or C–C bonds, indicating that all diamond surfaces remain intact, and no wear is generated at 1 GPa in the simulated time interval.

There are two critical factors leading to the formation of C–O–Si chemical bonds at the silica–diamond interface: (i) the presence of the carbonyl groups containing chemically active nonbridging oxygen atoms that are capable of capturing hydrogen atoms of the silanol groups, and (ii) the availability of un-terminated carbon atoms that can immediately form Si–O–C bonds following the OH bond dissociation. These two factors only coexist on C001-50%O and C110-50%O surfaces. For the *R*-C111-50%O, the oxygen atoms of the ether configuration terminate all active carbon sites of the topmost layer,^8^ resulting in the absence of the un-passivated carbon atoms and the less chemically active C–O–C configuration compared to the C=O one (Figure 1e). Similarly, on the C110-50%O surface, the combination of carbonyl and epoxy configurations involves more carbon atoms at the topmost layer compared to the C(001) surface,^46^ making the C110-50%O surface less active toward silica than C001-50%O surface. These observations also explain the absence of chemical bond formation at interfaces of the 100%O coverage systems, as no active carbon atoms are available on the surfaces. Consequently, the results clearly demonstrate that the full oxygen coverage of the diamond surface effectively prevents the chemical bond formation across the interfaces.

**Figure 3:**
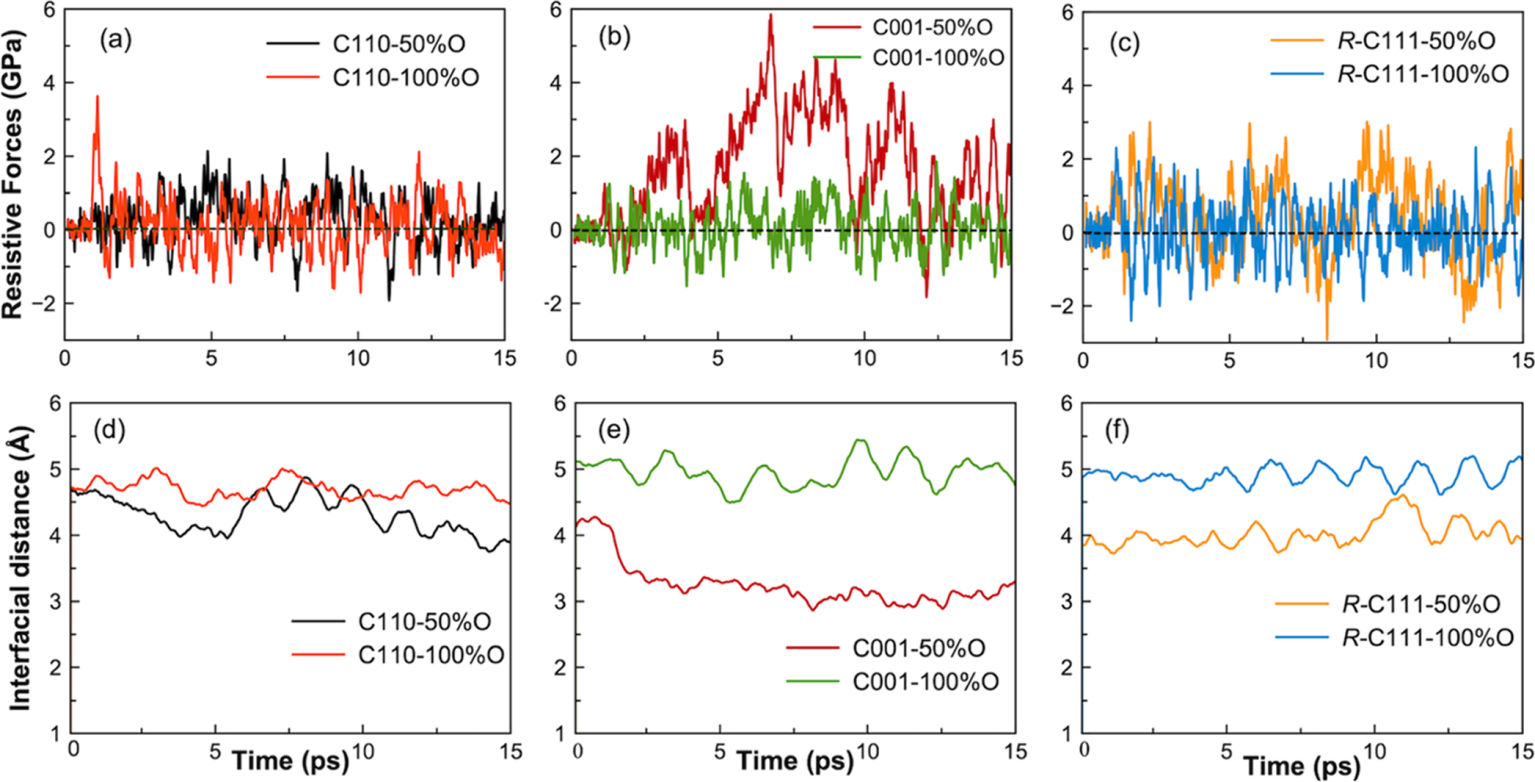
Time evolution of resistive forces per area (a–c) and interfacial distance (d–f) recorded during the tribological simulations of silica–diamond interfaces.

**Figure 4:**
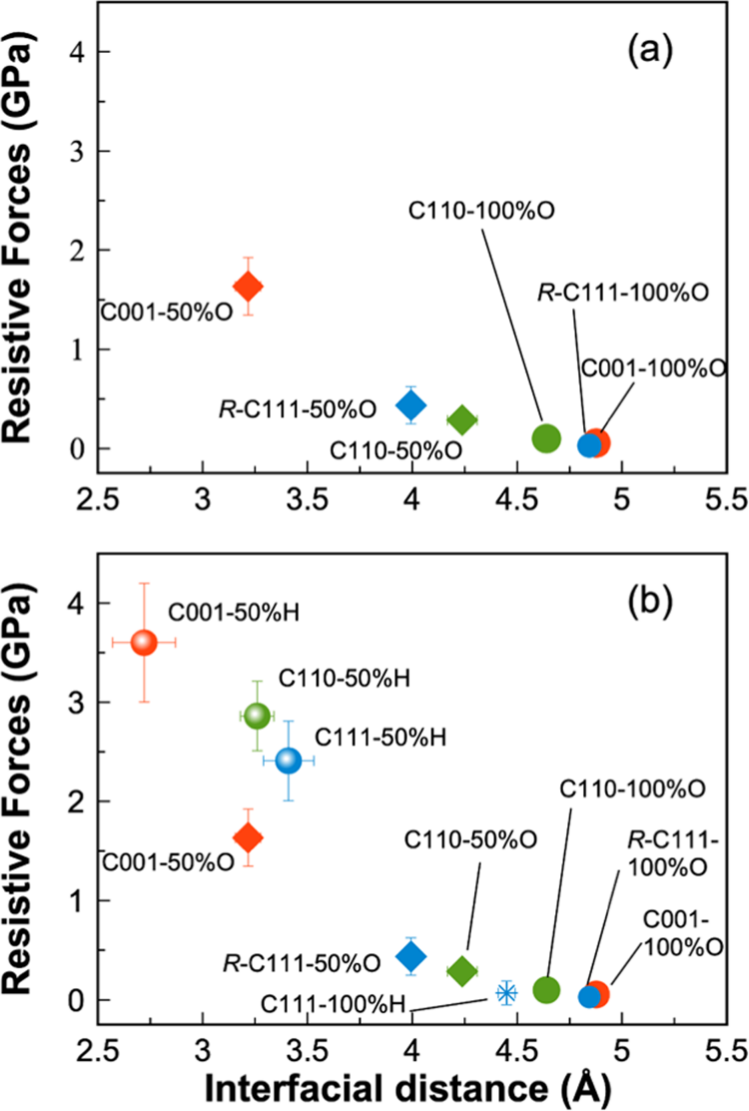
Mean resistive forces per area and interfacial distance of the silica sliding against O-terminated diamond (a) and the comparison of the mean resistive forces and interfacial distances in O-terminated and H-terminated diamond surfaces (b). Color assignment: C(001) (blue), C(110) (red), and C(111) (green). The data of the C001-50%H, C110-50%H, and C111-50%/100%H in (b) are collected from Cutini et al.^23^

The calculations of resistive stress and interfacial distances recorded during the simulation are presented in Figure 3, while the average values over the sliding period of 15 ps are reported in Figure 4 and Table 2. It clearly shows that distinct gaps ranging from 4 to 5 Å are maintained in five of the six simulation systems, except for the C001-50%O case. There is a correlation between the resistive force and the interfacial distance, i.e., the larger the interfacial gap, the lower the resistive force. Notably, the interfacial distances of approximately 5 Å which correspond to the lowest resistive forces, are consistently achieved in all of the systems with 100%O coverage. This suggests that the full oxygen coverage yields the most effective adhesion reduction, in accordance with the literature indicating that a passivation concentration exceeding 50% is necessary for friction reduction.^17^ The complete passivation of the surface is effective in preventing chemical bonds across the silica–diamond interfaces, thereby reducing adhesion and resistive forces. Conversely, the C001-50%O system exhibits the highest resistive forces, with the interfacial distance reduced to approximately 3.0 Å (Figure 3e). In this particular case, the formation of Si–O–C chemical bonds, as illustrated in Figure 2f–i, plays a key role in increasing adhesion and resistant forces.^47^ It is worth mentioning that the interfacial distance is calculated between the lowest Si atoms and the topmost carbon layer. Thus, an interfacial distance of at least ∼3.12 Å, which corresponds to the sum of the Si–O and C–O bond lengths, is necessary to establish chemical bonds across the interface.

**Table 2:** Mean Resistive Forces Per Area (*F_x_*, in GPa) and Interfacial Distance (Δ*Z*, in Å) of the Silica Sliding against O-/H-Terminated Surfaces

system	Δ*Z*	*F_x_*	system	Δ*Z*	*F_x_*	system^a^	Δ*Z*	*F_x_*
C110-50%O	4.238	0.29	C110-100%O	4.640	0.10	C110-50%H	3.26	2.86
C001-50%O	3.216	1.64	C001-100%O	4.877	0.06	C001-50%H	2.72	3.60
*R-*C111-50%O	3.994	0.44	*R-*C111-100%O	4.844	0.03	C111-50%H	3.41	2.41

a The data of the C001-50%H, C110-50%H, and C111-50% are collected from Cutini et al.^23^

A comparison of the resistive forces in O- and H-passivated systems is presented in Figure 4b. The full oxygenation of the diamond surfaces provides comparable friction reduction to the fully hydrogenated systems. Interestingly, for the half-coverage, oxygenation can provide even better friction reduction than hydrogenation, which is indicated by the lower resistive forces and higher interfacial distances obtained in O-passivated systems. The larger interfacial gaps achieved in O-terminated diamond can be attributed to the larger size of oxygen, resulting in a greater hindrance effect compared to that of hydrogen. Another reason is that the presence of oxygen atoms at the diamond and silica surfaces promotes electrostatic repulsion between the two surfaces, thus keeping the two surfaces apart. It has been shown that the repulsion between two sliding surface is possible and play a key role in reducing friction in passivated diamond.^48^ This electrostatic repulsion arises from the negatively charged oxygen atoms as indicated by the Bader charges reported in Table 3. In particular, the average Bader charges of oxygen range from −0.65 to −0.95 *e* on diamond surfaces, and −1.53 *e* in silica. These negative charges contribute to the repulsive interaction between the opposing surfaces. Whereas, in H-terminated systems, the attraction between oxygen-rich silica and hydrogen atoms on the diamond surface keeps the two surfaces at close distances. Therefore, full oxygenation of the diamond surface can provide even lower resistive forces compared to the corresponding hydrogenation, highlighting the benefits of oxygenation in friction reduction.

**Table 3:** Average Bader Charges (*e*) of Atoms in Diamond Surfaces and Silica^a^

	C(110)			C(001)			*R*-C(111)			
system	50%O	100%O	50%H	50%O	100%O	50%H	50%O	100%O	50%H	silica
C/Si^b^	0.07	0.11	0.03	0.08	0.11	0.03	0.07	0.14	0.03	+3.16
C(=O)/C(−H)	+1.02	+1.01	–0.03	+0.89	+0.71	–0.01		+0.91	–0.02	
C(−O–C)	+0.39	+0.37					+0.35			
O(=C)/O(−Si)^b^	–0.95	–0.91		–0.87	–0.65			–0.90		–1.53
O(−C)	–0.80	–0.78					–0.72			
H(−C)/H(−O)^b^			+0.06			+0.06			+0.05	+0.67

a The averages are calculated considering the same atomic types in the systems.

b Only in silica.

**Figure 5:**
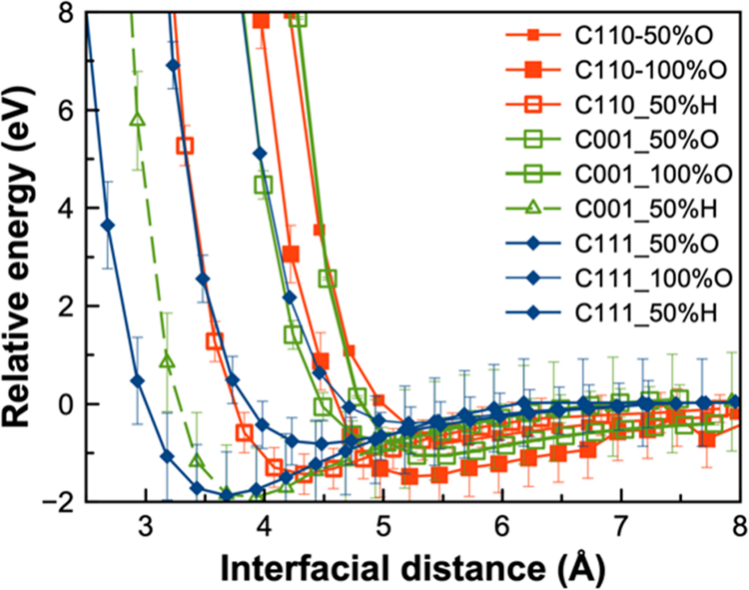
P-PESs of the silica–diamond systems as a function of interfacial distance. The interfacial distance is measured from the average *z* coordinates of the lowest Si atoms to the highest C atoms.

To provide more insights into the nature of silica–diamond interactions in O- and H-terminated systems, we performed the calculations of P-PESs for the six systems studied in this work, along with three H-terminated systems from our previous work.^23^ As depicted in Figure 5, the hydrogen-terminated systems (green curves) exhibit deeper minima at the shorter separation compared to all oxygen-terminated systems. This can be due to the charge distribution that promotes the hydrogen interaction between the H-passivated diamond and the O-rich silica and attracts the two surfaces at a closer distance. This is unlike the full H-coverage surfaces where all sp^2^-carbon atoms are terminated, the repulsion at a short distance (less than 2.5 Å) can be beneficial to produce ultralow friction.^49,50^ While the H–H repulsion can also be present in this system due to the H from the silanol groups, it can only be effective at short distances which allow interfacial bonding to occur. Meanwhile, the minima of oxygen-terminated systems (blue and red curves) shift to higher interfacial distances, suggesting that the repulsion is dominated by the oxygen layer. The higher repulsion in oxygen-terminated systems when two surfaces come closer helps to maintain a clear gap between silica and diamond surface, thus reducing resistive forces and friction.

### 3.3 Initial Stage of Diamond Wear in Harsh Conditions

Hash conditions of 10 GPa load and 600 K are applied to facilitate the formation of wear in silica–diamond systems within the simulation time interval. First, all of the six simulation systems were relaxed at 10 GPa to assess their reactivity under this severe condition. The optimized structures at 10 GPa are shown in Figure S3. The result indicates that only two systems, C110-50%O and C001-50%O, among the six systems, show Si–O–C chemical bonds across the interface. This result indicates that C110-50%O and C001-50%O are the most reactive surfaces under the harsh conditions. The presence of the Si–O–C interfacial bonds is essential to initiate the wear of diamond surfaces, making the C110-50%O and C001-50%O the most wearable surfaces among the six surfaces.^26^ In addition, previous studies have reported that the C(111) surface is the hardest facet of diamond to be polished.^51^ Therefore, in order to gain insights into the formation wear and its mechanisms, our AIMD simulations in the harsh conditions are focused on the C110-50%O and C001-50%O systems.

**Figure 6:**
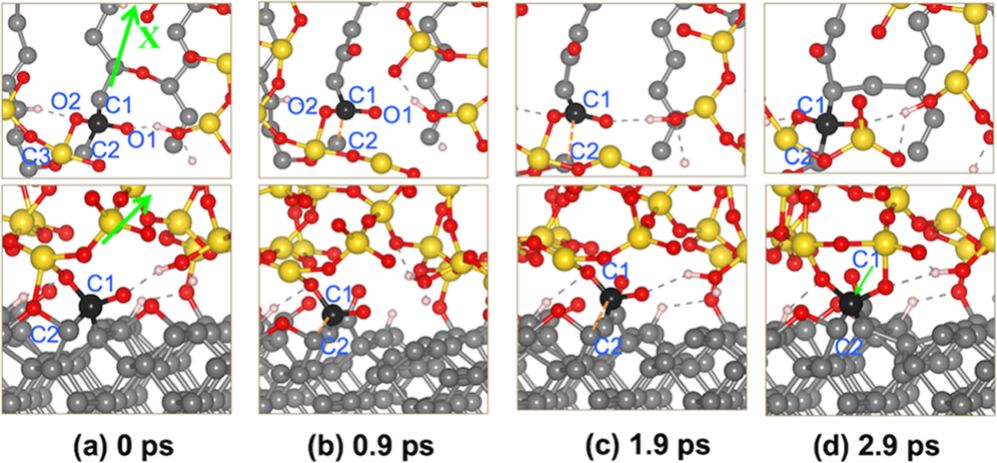
Top (first row) and side (second row) views of the C–C bond dissociation in the silica–C110-50%O system under the harsh conditions. The bold ball represents the detached carbon atom. Only one topmost layer of the C(110) surface and one bottommost layer of the silica slab are shown in the top views. (a) Initial configuration containing C1 bonded to two O atoms, (b, c) C1–C2 bond dissociation, and (d) C1–C2 bond recombination.

As can be seen in Figure 6, in the C110-50%O system, after the relaxation at 10 GPa, the bonding of oxygen atoms to C1 and C2 makes the C1–C2 bond weaker followed by an increased bond length to 1.67 Å (Figure 6a). This bond length is larger than the average value of 1.35–1.54 Å measured for other C–C bonds of zigzag chain on the top layer.^26^ The C–C bond rupture occurs at 0.95 ps of the equilibrium process. After sliding for ∼0.9 ps, the C1–C2 distance reaches a value of 2.51 Å (Figure 6c), indicating the complete dissociation of the C1–C2 bond. However, under the applied load, C1 and C2 recombine at ∼1.9 ps. The further sliding predominantly involves the dissociation of Si–O/C–O bonds, while the C1–C2 bond remains intact.

**Figure 7:**
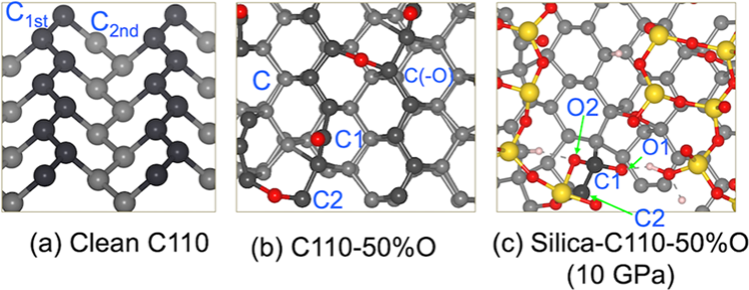
Top (darker balls) and second top layers of the clean C(110) surface (a), C110-50%O (b), and silica–C110-50%O optimized under 10 GPa (c). This figure shows the atom labels used for the BOP results in Table 4.

In order to understand the nature of the bonding in the silica–diamond system, the bond overlap population was estimated to compare the bond strength of the C–C bonds in the clean C(110), 50%O coverage C(110), and silica–C110-50%O at 10 GPa.^45^ The results reported in Table 4 show that the C_1st_–C_1st_ bonds (the darker atoms in Figure 7) are stronger than the C_1st_–C_2nd_ bonds. This is consistent with what has been reported by Peguiron et al. indicating that the C–C bond dissociation primarily occurs at the C–C bonds which connect the carbon atoms of the zigzag chain and those of the underlying layer.^26^ When the surface is oxidized, the BOP values of the C_1st_–C_1st_ and C_1st_–C_2nd_ bonds are reduced from 0.41 to 0.37 *e*, indicating that the C–C becomes weakened under oxygenation.^52^ However, the value is considerably higher than that of Si–O bonds (0.27 *e*). As a result, the bond breaking frequently occurs at the Si–O sites during sliding. When C is bonded to two O atoms (C1), the BOP of the C1–C2 bond drops significantly from 0.53 *e* on the clean surface to 0.36 *e* on the oxidized C(110), and further reducing to 0.32 *e* in the silica–C(110) at 10 GPa. Consequently, the C–C bond breaking was observed during the AIMD simulation at 10 GPa (Figure 6b). Therefore, it is necessary to have C atoms bonded to two oxygen atoms to weaken the C–C bonds, as in the C1–C2 case. This is consistent with our recent density functional theory (DFT) calculation suggesting that wear can appear on the C(110) surface at the C=O double bond or −O–C–O–Si bidentate structure.^53^ In this work, the −O–C–O–Si bidentate structure is built by one oxygen atom of silanol to the C(=O) atom of the diamond. Another potential scenario involves two oxygen atoms in a geminal silanol with an un-passivated carbon atom. However, the un-passivated carbon is less presentative in the oxygenated surface., while geminal silanols are rarely or even not detected in silica.^40^ Thus their contribution to the formation of the −O–C–O–Si structure is neglectable, and the absence of geminal silanols in our silica model does not significantly impact the established wear mechanism of oxygenated diamond.

**Table 4:** Bond Overlap Population of C–C and Si–O Bonds^a^

bond/system	clean C(110)	C110-50%O	C110-50%O (10 GPa)
C_1st_–C_1st_	0.53	0.45	0.43
C_1st_–C_2nd_	0.41	0.37	0.37
C–C		0.46	0.45
C–C(−O)		0.39	0.39
C1–C2	0.53	0.36	0.32
Si–O			0.27

a C_1st_ and C_2nd_ are carbon atoms of the first and second layers of the C(110) surface, respectively, as shown in Figure 7a. C is a carbon atom on the top layer of the C(110) surface, and C(−O) is the carbon terminated by oxygen on the C110-50%O surface shown in Figure 7b. C1 and C2 are the two carbon atoms shown in Figure 7c. The BOP is calculated as the average over atoms of the same kind in each considered system.

Snapshots of the C001-50%O system during sliding in the harsh conditions are reported in Figure S4. It is clearly indicative that although Si–O–C bond formation occurs across the C(001)–silica interface, the lateral displacement leads to the dissociation of Si–O/C–O bonds, and the C(001) surface remains unaffected. This is consistent with the DFT calculation showing C(001) surface is less vulnerable to wear than C(110) one.^53^

In summary, the C–C bond dissociation on the diamond surfaces occurs in subsequent steps: (1) the O–H bond of the silanol group is dissociated, leaving a Si–O dangling bond, (2) the Si–O dangling bond is attached to the diamond surface, forming Si–O–C bridge, (3) when the C atom forms covalent bonds with two O atoms, it becomes weakened and the C–C bond breaking occurs.

### 3.4 Evolution of Atomic Wear in the Presence of Passivating Species

**Figure 8:**
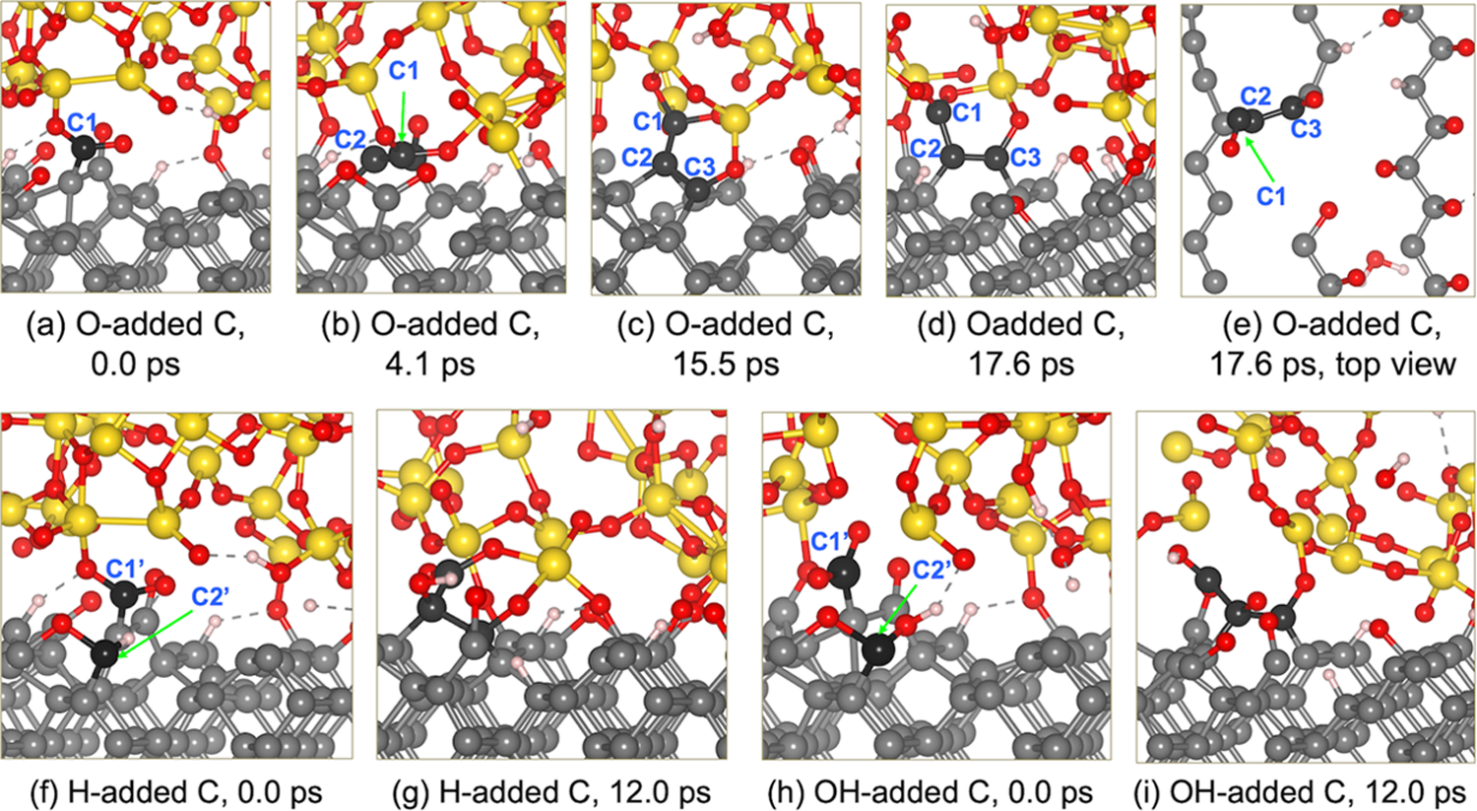
Formation of wear at the diamond–silica interface when O, H, and OH fragments terminate the C dangling bond (Movie S4). (a, f, h) Initial structure when the detached C is terminated by O/H/OH, respectively. (b–d) C–C bond dissociation to form a carbon chain under sliding. (e) Top view of the formation of the carbon chain. (g, i) Formation of the carbon chains during sliding when the detached C is terminated by H and OH, respectively.

Small molecules such as O_2_, H_2_, or H_2_O abundant in the working environment can diffuse into the sliding contact. When a C–C bond is broken, these small molecules could passivate the newly formed dangling bond. To investigate this possibility, we performed DFT calculations on the adsorption and dissociation of O_2_, H_2_, and H_2_O at the C–C breaking site on C110-50%O. The results indicate that the dissociation is thermodynamically favorable with reaction energies of −2.37, −3.08, and −2.14 for O_2_, H_2_, and H_2_O, respectively (Figure S5). The kinetic barriers of O_2_, H_2_, and H_2_O dissociation on clean C(110) surface are around 0.75–1.27 eV,^24^ and similar reaction barriers can be expected on C110-50%O. Under harsh tribological conditions, these barriers can be overcome to facilitate the molecular dissociation, suggesting that the dangling carbon created by the C–C bond breaking can be saturated by O, H, and OH fragments from the dissociated molecules. Selected configurations explored during the sliding of O-added, H-added, and OH-added silica–C(110) systems under 10 GPa load are reported in Figure 8.

When the carbon atom (C2) of the dangling bond is terminated, the bond recombination occurring in Figure 6d is not observed, and the sliding under 10 GPa leads to the dissociation of additional C–C bonds. Following the C1–C2 bond breaking, the bonds between C2 and C3 are also broken at 4.1 and 17.6 ps, respectively. Carbon atoms C1, C2, and C3 move upward and form chemical bonds with the silica. The one-by-one C–C bond dissociation results in the breaking of the zigzag chain on the top layer, leaving a defective space on the surface as shown in Figure 8e. Thus wear is found in the form of carbon chains, which was also found in diamond polishing by silica in aqueous H_2_O_2_.^53^ Similar C–C bond breaking is observed when the detached carbon is terminated by H and OH groups at the C2′ site (Figure 8f,h). The carbon chains are also observed as shown by snapshots at 12 ps (Figure 8g,i). These findings suggest that the presence of environmental species plays an important role in facilitating wear. In particular, the adsorption of the atmospheric molecules not only weakens the C–C bonds on the diamond surface but also terminates the dangling sites produced from C–C bond breaking, thus preventing the bond recombination and promoting wear.

## 4 Conclusions

Silica/diamond interfaces are present in many tribological applications such as micro-electromechanical systems and atomic force microscopes. The friction behavior and the atomistic wear mechanism of silica sliding against oxygenated diamond surfaces have been studied by AIMD simulations accompanied by atomic and electronic structure analyses. The obtained results can be summarized as follows:

1. The full coverage of diamond with oxygen is highly effective to reduce adhesion and the formation of chemical bonds across the silica–diamond interfaces. The outcome holds true for all three C(110), C(001), and *R*-C(111) surfaces at both 1 and 10 GPa loads. The resistant forces in the case of the full O coverage are even lower than those of the full H-coverage. This is due to the larger steric hindrance of oxygen and its electrostatic repulsion with the silica surface. The situation drastically changes for the lower O coverage of different surface orientations. In particular, at 50% coverage, we observe the formation of Si–O–C bonds across the interface for the C(001) surface.
2. Under the harsh working conditions, chemical bonds are established at the interfaces of silica and half-passivated diamond surfaces except for the case of the *R*-C111-50%. However, the formation of Si–O–C bonds is not enough to induce the C–C bond breaking, which occurs only in the C110-50%O system when the C–C bond is weakened by the chemical bonds forming by two oxygen atoms at the same C site.
3. The recombination of the C–C broken bond can be prevented by the dissociative adsorption of passivating molecules present in the environment. The wear mechanism is then dominated by the detachment of small carbon chains.

Our simulations indicate that full oxygenation is an effective technique for friction reduction and reveal the mechanical–chemical conditions to explain the wear formation in diamond–silica systems at partial O coverage, which helps answer the question of how silica can polish diamond.

## Supporting Information Available

Sliding of silica against 50%O diamond surfaces at 1 GPa (Movies S1–S3) and the sliding of silica against C110-50%O at 10 GPa (Movie 4) (ZIP)
Top and side views of oxidized diamond surfaces, relaxed structures of silica–diamond systems at 1 and 10 GPa, and structures of molecular and dissociative adsorption of O_2_/H_2_/H_2_O on C110-50%O (PDF)
