# Supplementary material for: Nanotribological Properties of Oxidized Diamond/Silica Interfaces: Insights into the Atomistic Mechanisms of Wear and Friction by Ab Initio Molecular Dynamics Simulations
Source: ACS Appl Nano Mater. 2023 Sep 4;6(18):16674–83. doi: 10.1021/acsanm.3c02881 (PMC10520978; doi:10.1021/acsanm.3c02881)
Supplement: Supplementary file 2 — an3c02881_si_002.pdf [file an3c02881_si_002.pdf]

Supporting Information

Nanotribological Properties of Oxidized Diamond/Silica Interfaces: Insights into the Atomistic Mechanisms of Wear and Friction by *Ab Initio* Molecular Dynamics Simulations

Huong Thi Thuy Ta[1], Nam Van Tran[1], and Maria Clelia Righi[1]*

[1]Department of Physics and Astronomy, University of Bologna, 40127 Bologna, Italy

Corresponding Author:

M.C. Righi – E-Mail: clelia.righi@unibo.it, Department of Physics and Astronomy, University of Bologna, 40127 Bologna, Italy.

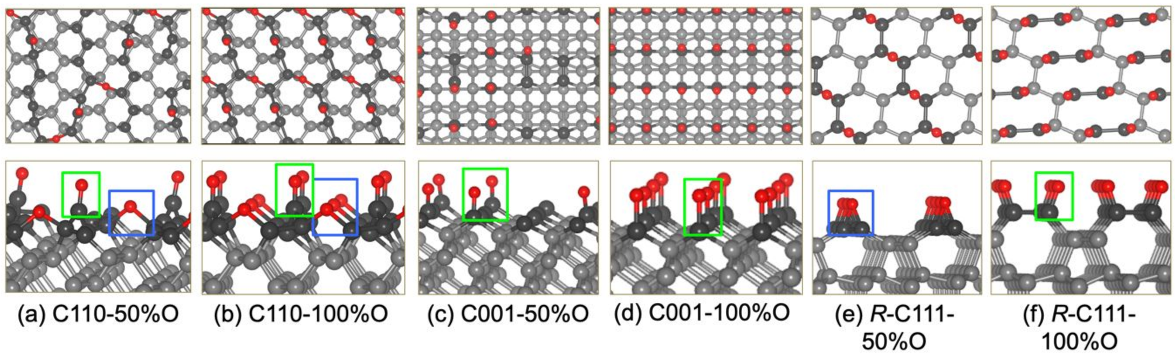

Figure S1. Top (first row) and side (second row) views of the optimized structures of the 50% and 100% oxygen terminated C(110), C(001), and R-C(111) surfaces. The darker balls show the carbon atoms at the top layer. The green and blue boxes mark the carbonyl and ether configurations of the oxidized diamond surfaces.

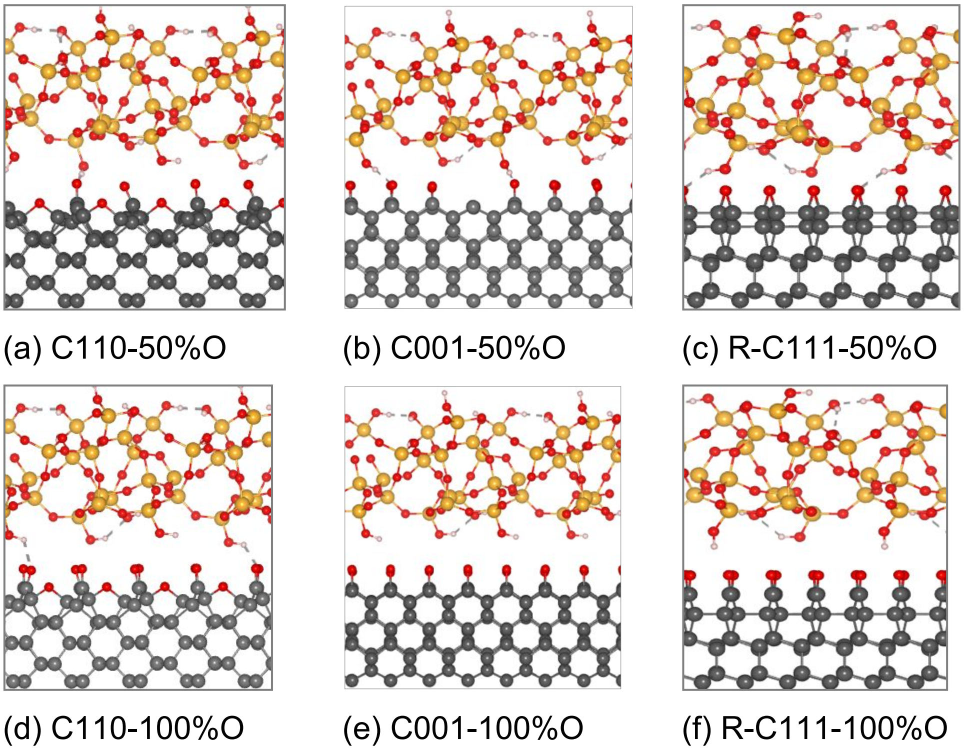

Figure S2. Stable configurations of silica-diamond systems relaxed at 1 GPa.

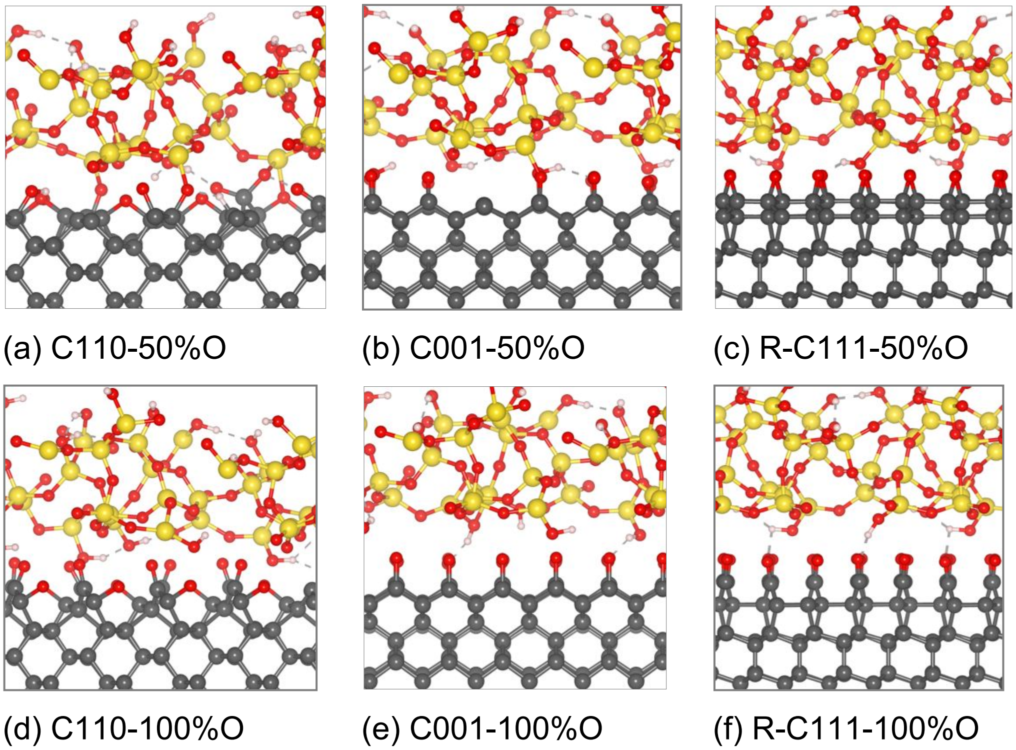

Figure S3. Stable configurations of silica-diamond systems relaxed at 10 GPa.

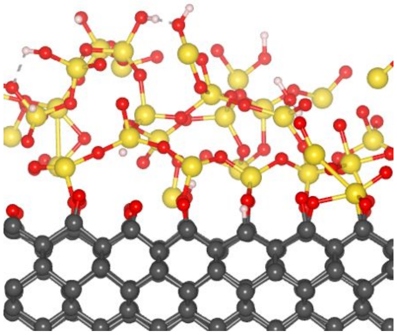

Figure S4. Structures of the silica-C001-50%O system during the sliding at 10 GPa and 600 K for 8 ps.

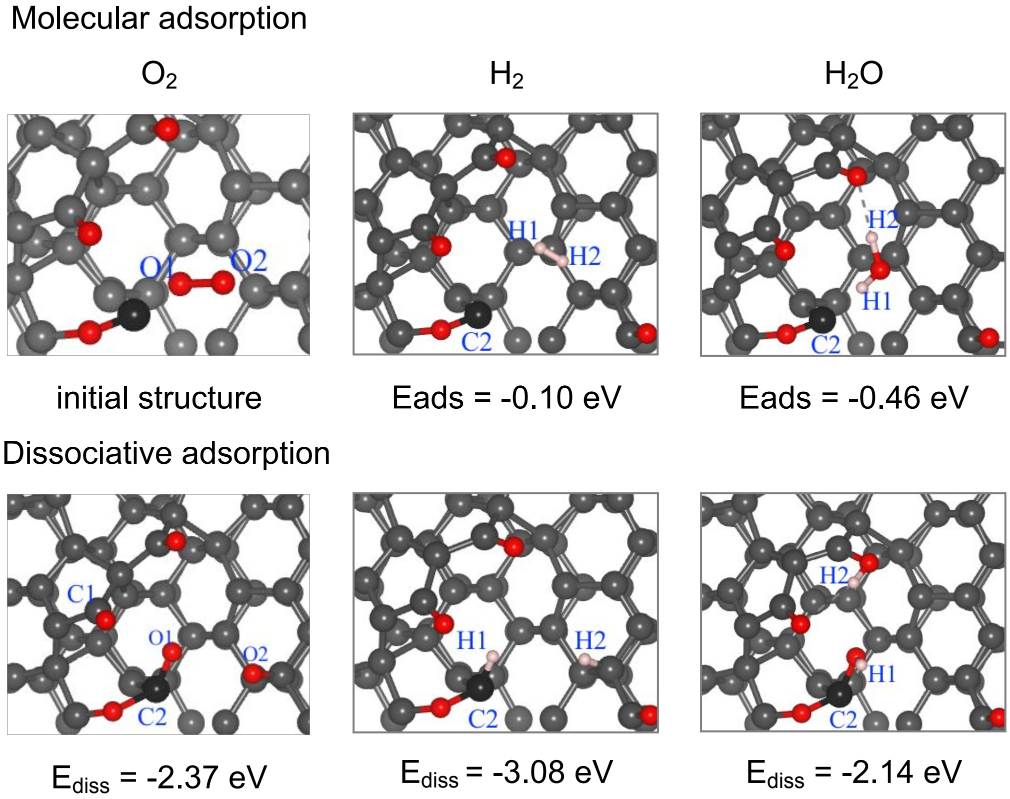

Figure S5. Molecular and dissociative adsorption of O_2, H_2, and H_2O on the C110-50%O surface when the C1-C2 bond was detached.
